# Heart Failure Remote Monitoring: Evidence From the Retrospective Evaluation of a Real-World Remote Monitoring Program

**DOI:** 10.2196/jmir.4417

**Published:** 2015-04-22

**Authors:** Stephen Agboola, Kamal Jethwani, Kholoud Khateeb, Stephanie Moore, Joseph Kvedar

**Affiliations:** ^1^Partners Healthcare Center for Connected HealthConnected Health InnovationBoston, MAUnited States; ^2^Massachusetts General HospitalBoston, MAUnited States; ^3^Harvard Medical SchoolBoston, MAUnited States; ^4^Northeastern UniversityBoston, MAUnited States

**Keywords:** heart failure, telemonitoring, remote monitoring, self-management, hospitalizations, mortality

## Abstract

**Background:**

Given the magnitude of increasing heart failure mortality, multidisciplinary approaches, in the form of disease management programs and other integrative models of care, are recommended to optimize treatment outcomes. Remote monitoring, either as structured telephone support or telemonitoring or a combination of both, is fast becoming an integral part of many disease management programs. However, studies reporting on the evaluation of real-world heart failure remote monitoring programs are scarce.

**Objective:**

This study aims to evaluate the effect of a heart failure telemonitoring program, Connected Cardiac Care Program (CCCP), on hospitalization and mortality in a retrospective database review of medical records of patients with heart failure receiving care at the Massachusetts General Hospital.

**Methods:**

Patients enrolled in the CCCP heart failure monitoring program at the Massachusetts General Hospital were matched 1:1 with usual care patients. Control patients received care from similar clinical settings as CCCP patients and were identified from a large clinical data registry. The primary endpoint was all-cause mortality and hospitalizations assessed during the 4-month program duration. Secondary outcomes included hospitalization and mortality rates (obtained by following up on patients over an additional 8 months after program completion for a total duration of 1 year), risk for multiple hospitalizations and length of stay. The Cox proportional hazard model, stratified on the matched pairs, was used to assess primary outcomes.

**Results:**

A total of 348 patients were included in the time-to-event analyses. The baseline rates of hospitalizations prior to program enrollment did not differ significantly by group. Compared with controls, hospitalization rates decreased within the first 30 days of program enrollment: hazard ratio (HR)=0.52, 95% CI 0.31-0.86, *P*=.01). The differential effect on hospitalization rates remained consistent until the end of the 4-month program (HR=0.74, 95% CI 0.54-1.02, *P*=.06). The program was also associated with lower mortality rates at the end of the 4-month program: relative risk (RR)=0.33, 95% 0.11-0.97, *P*=.04). Additional 8-months follow-up following program completion did not show residual beneficial effects of the CCCP program on mortality (HR=0.64, 95% 0.34-1.21, *P*=.17) or hospitalizations (HR=1.12, 95% 0.90-1.41, *P*=.31).

**Conclusions:**

CCCP was associated with significantly lower hospitalization rates up to 90 days and significantly lower mortality rates over 120 days of the program. However, these effects did not persist beyond the 120-day program duration.

## Introduction

Despite the advances made in the management of heart failure, the burden of disease due to heart failure still remains unacceptably high. Prevalence is projected to increase by 46% from 2012-2030 [1]. Data suggest that hospitalization and mortality rates did not change much from 2000-2010 [2]. Heart failure contributed to about 1 in 9 causes of death in 2009, and it is estimated that about half the 825,000 new cases of heart failure diagnosed annually will die within 5 years of diagnosis [2]. The cost of heart failure is also projected to increase by about 127% from the estimated US $30.7 billion spent in 2012 to US $69.7 billion in 2030 [1].

Given the magnitude of this problem, multidisciplinary approaches in the form of disease management programs and other integrative models of care are recommended to optimize treatment outcomes [3]. Remote monitoring, either as structured telephone support or telemonitoring or a combination of both, is fast becoming an integral part of many disease management programs [4-6]. The main strategy is to either provide some sort of education around self-care, or monitor patients for early detection of heart failure decompensation and intervention. Outcomes reported in the current heart failure telemonitoring literature have varied not only based on the type of technology used but also on intensity, complexity of intervention, speed of clinical decision-making, and patient-clinician factors. Telemonitoring approaches vary from simple, noninvasive monitoring of physiologic parameters like heart rate, blood pressure, and weight, to more advanced and invasive approaches like monitoring of intracardiac pressures [7].

An ideal heart failure remote monitoring approach would be one that empower patients for self-care—knowing and able to perform day-to-day care processes, able to early identify symptoms of worsening of disease, and initiating appropriate action. This can be achieved by a care model that incorporates regular out-patient clinic follow-up with ongoing education (provided at clinic visits or via telemonitoring), objective monitoring of clinical condition, and provision of timely feedback. The telemonitoring program at the Partners HealthCare, Center for Connected Health, Connected Cardiac Care Program (CCCP), is one of such programs that model this approach.

**Figure 1:**
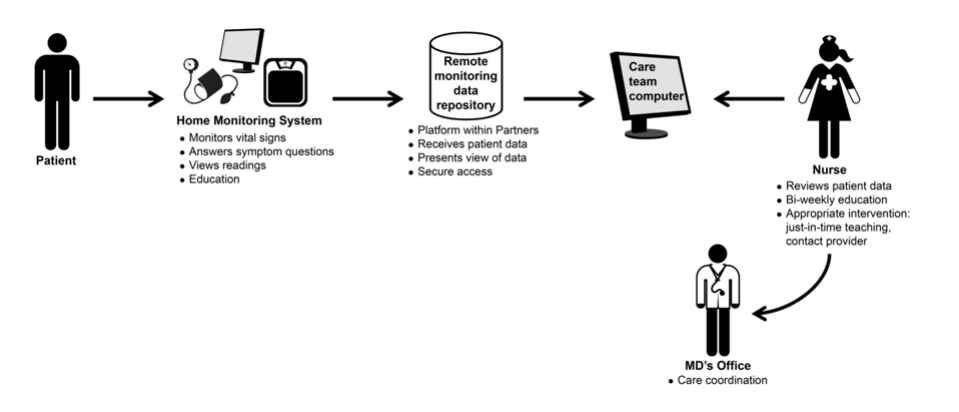
The Connected Cardiac Care Program.

CCCP is a 4-month home telemonitoring and education program designed to improve self-management in heart failure patients at risk for hospitalization within the Partners HealthCare network of hospitals. Participants monitor relevant physiologic parameters (blood pressure, heart rate, weight, and blood oxygen saturation) and answer questions on heart failure–related symptoms on a touch-screen computer on a daily basis (Figure 1). The remote monitoring equipment included ViTel Net and devices approved by the Food and Drug Administration: a UA 767PC Turtle 400 monitor, a Life-Source digital weight scale, an A&D blood pressure cuff and meter, and a BCI pulse oximeter device (UC-321PBT). Measurements and responses to symptom questions are transferred securely to a remote monitoring database where the records are reviewed by telemonitoring nurses. Participants also receive structured biweekly telephone-based education sessions over an 8-week period. Patient education covered a variety of topics including diet, physical activity, importance of daily measurements, recognizing symptoms of disease decompensation, and medication adherence. In addition to the structured educational sessions, they received “just-in time” teaching, that is, unscheduled education done to intervene when the remote monitoring nurses observe that measurements fall outside the set baseline range customized for each participant by their physicians or at the onset of new symptoms.

This study aims to evaluate the effect of a heart failure telemonitoring program, Connected Cardiac Care Program (CCCP), on hospitalization and mortality in a retrospective database review of medical records of patients with heart failure receiving care at the Massachusetts General Hospital (MGH). While the heart failure remote monitoring literature is replete with studies in controlled settings, there is a dearth of literature reporting on the evaluation of real-world heart failure remote monitoring programs. In addition, by following up with patients after disenrollment from the CCCP program, this study sheds some light on the downstream impact of completely taking patients (who have built disease self-efficacy skills) off remote monitoring programs.

## Methods

### Overview

This study is a retrospective analysis to evaluate the effect of CCCP on clinical outcomes in patients enrolled in the program in a 1:1 match cohort study. Matching on potential confounders is a methodology that is commonly adopted to increase efficiency [8,9]. It is generally suitable for situations where the investigators have access to large population data sources [9].

CCCP participants were compared with control patients, within the same health care system, not enrolled in the CCCP. The control patients received the usual standard of care at MGH. A 1:1 individual matching was done to identify controls for each CCCP participant by selecting a control patient that had a hospitalization within 30 days of the corresponding CCCP’s patient index hospital admission. Every patient enrolled in the CCCP program must have an index hospitalization. The index admission is the last heart failure–related hospitalization a patient must have prior to enrollment in the CCCP. Other matching parameters are age ±2 years, race, and gender. We used the matching without replacement method. In this method, once a matched patient from the control population is selected, they are no longer eligible for subsequent selection. The best matched control was selected to maximize the precision of the analysis. Gender and race were the first considerations in selecting the best match, followed by the nearest age and index admission date in that order.

### Study Population

All subjects included in this study are patients with a diagnosis of heart failure receiving care at MGH. Eligible participants were English-speaking heart failure patients, who had a Partners HealthCare primary care provider or cardiologist that utilized the electronic medical record. They were also required to have a hospital admission to a Partners HealthCare hospital to be eligible to participate in the CCCP. Eligibility requirement for enrollment in the program included that patients must have a diagnosis of heart failure, must have an MGH care provider, and must have been flagged as having a high risk for readmission and referred into the program by their care provider. Patients with end-stage renal disease, on any chemotherapeutic medication, or having had any organ transplant were excluded from participation.

We conducted a retrospective review of the remote monitoring database (RMDR) to identify participants enrolled in the CCCP between Jan. 1, 2008, and Aug. 31, 2012. The RMDR is a secured database housed within the Partners HealthCare firewall where connected health data are processed and stored. From the RMDR, we also collected program information of participants. This information included program start of care and end of care dates. The list of identified program participants was sent to the Partners HealthCare’s Research Patient Data Repository (RPDR) to access participants’ full clinical data and also to identify eligible match controls. The RPDR is a large clinical data registry that gathers medical records from various hospital systems and stores them in a central location [10]. We identified control patients, not participating in the CCCP but receiving care at MGH, with any heart failure-related International Classification of Disease, Ninth Revision, Clinical Modification (ICD-9-CM) and with similar CCCP eligibility requirements. We excluded CCCP patients whose enrollment date in the program was before the year of interest (2008), without documented index hospital admission before CCCP enrollment. We also excluded CCCP patients (and corresponding matched controls) that were enrolled in the program for less than 1 week. Control patients (and corresponding CCCP patient) with incomplete clinical data, those not meeting CCCP entry criteria, and those who died prior to the CCCP enrollment date of corresponding CCCP match were also excluded from analyses.

### Source of Data

The primary source of data for this study is the RPDR. The detailed dataset from the RPDR contained demographic and clinical information of patients including age, gender, race, diagnoses, hospital visits, clinical notes, and vital status. All diagnoses were carefully verified in the electronic medical records to limit misclassification of disease status due to data coding errors. We collected and reviewed all-cause hospitalization and mortality data of CCCP patients and their corresponding controls starting from 120 days before enrollment in the CCCP program and up until 1 year after the CCCP enrollment date. The study was approved by the Partners HealthCare Human Research Committee.

### Outcome Measures

Outcomes were classified based on follow-up times. The primary outcomes were the effects of the intervention assessed during the program duration of 4 months. The primary effects at 30 days, 60 days, 90 days, and at 4 months were on mortality and hospitalizations.

Secondary outcomes were the cumulative effects of the intervention assessed over a 1-year follow-up period from the date of program enrollment, that is, following up participants for an additional follow-up time of 8-months following program completion. The secondary effects evaluated were on mortality, hospitalizations, risk for multiple hospitalizations, and length of hospital stay.

### Statistical Analysis

Baseline data were summarized using descriptive statistics: means and standard deviation for continuous data with normal distribution, medians for skewed data, and percentages for categorical data. We examined group differences using the *t* test or the Wilcoxon-Mann-Whitney test for continuous data and chi-square tests for categorical data. We also assessed baseline hospitalization rates 120 days prior to CCCP enrollment to see if differences existed in hospitalization rates across both groups prior to follow-up. Cumulative survival curves for time-to-event analyses were constructed by the Kaplan-Meier method. The Cox proportional hazard model using time to death and hospitalization as endpoints was used to estimate hazard ratios (HR). Multiple hospitalizations were accounted for in the model. All analyses were stratified on the matched pairs [8], and we also adjusted for age, gender, race, ejection fraction, and New York Heart Association classification (NYHA). A two-sided *P*<.05 was considered as significant. All analyses were performed using data analysis and statistical software, STATA 12 version.

## Results

### Sample

**Figure 2:**
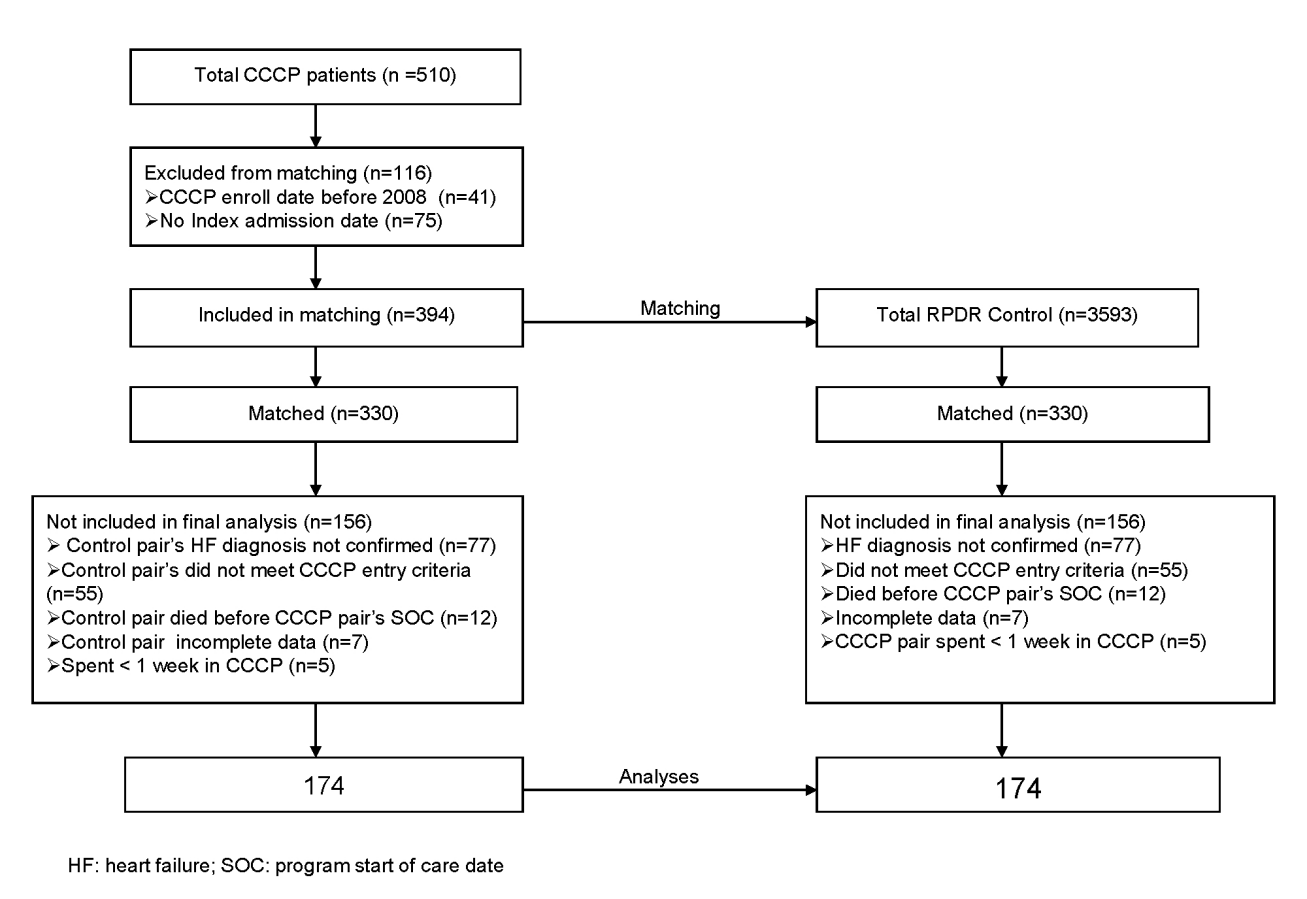
The sample selection process.

Figure 2 depicts the sample selection process. A total of 510 patients were enrolled in the CCCP from Jan. 1, 2008, to Aug. 31, 2012. Of these, 116 patients were excluded from matching. A total of 348 patients, 174 enrolled in CCCP and 174 match controls, were included in the final analysis.

The majority of the sample population were Caucasian males with an average age of 77 years in both groups, but they differed by marital status. The median duration of follow-up, 365 days, was similar in both groups. The baseline rates of hospitalization, 120 days prior to CCCP enrollment, did not differ significantly in the 2 groups (HR=1.02, 95% CI 0.83-1.24, *P*=.87). All patients were followed up for a maximum duration of 1 year from the period of program enrollment for CCCP patients and their corresponding controls. All baseline characteristics are summarized in Table 1.

**Table 1:** Baseline characteristics.

Characteristics		CCCP (n=174)	Control (n=174)	*P* value
Male, n (%)		102 (58.62)	102 (58.62)	
Age in years, mean (SD)		76.66 (10.71)	76.76 (10.71)	
Race (white), n (%)		158 (90.80)	158 (90.80)	
Ejection fraction, mean (SD)		49.17 (18.48)	50.66 (16.76)	.43
**NYHA class, n (%)**				.02
	I	5 (2.87)	15 (8.62)	
	II	116 (66.67)	125 (71.84)	
	III	49 (28.16)	32 (18.39)	
	IV	4 (2.30)	2 (1.15)	

### Primary Outcome

In the model accounting for multiple events, the hospitalization rate in both groups was similar at baseline (HR=1.02, 95% CI 0.83-1.24, *P*=.87). However, after 30 days of enrollment in the program, this rate decreased significantly in the CCCP group compared to the control group (HR=0.52, 95% CI 0.31-0.86, *P*=.01). This differential effect remained consistent throughout the duration of the program: at 60 days (HR=0.66, 95% CI 0.44-0.99, *P*=.05), at 90 days (HR=0.64, 95% CI 0.44-0.99, *P*=.02), and at the end of the 4-month program (HR=0.74, 95% CI 0.54-1.04, *P*=.06). Similar beneficial effect was observed on the mortality rate, which was significantly lower in CCCP patients compared with the control group (HR=0.33, 95% CI 0.11-0.97, *P*=.04) at the end of the program. The number of hospitalization and death events and corresponding rates measured at various points during the follow-up period are reported in Table 2.

**Table 2:** All-cause hospitalizations and mortality.

Follow-up		CCCP events, n	Control events, n	HR (95% CI)	*P* value
**All-cause hospitalizations during and after the program**					
	120 days before CCCP	220	209	1.02 (0.83-1.24)	.87
	30 days	24	49	0.52 (0.31-0.86)	.01
	60 days	43	68	0.66 (0.44-0.99)	.05
	90 days	56	87	0.64 (0.44-0.99)	.02
	120 days (CCCP ends)	75	97	0.74 (0.54-1.02)	.06
	1 year	180	151	1.12 (0.90-1.41)	.31
**All-cause mortality during and after the program**					
	120 days (CCCP ends)	5	12	0.33 (0.11-0.97)	.04
	1 year	22	31	0.64 (0.34-1.21)	.17

### Secondary Outcomes

Outcomes did not differ significantly by group over the additional 8 months of follow-up (ie, the course of the 1-year follow-up period from program enrollment). Of the 174 CCCP patients, 47% had at least one all-cause hospitalization over the 1-year follow-up period compared with 46% in controls (relative risk [RR]=1.03, *P*=.83) (Table 3). The risk for more multiple hospitalizations was higher in the CCCP group (25%) in comparison with controls (20%) but was not statistically significant (RR=1.29, *P*=.20). Additionally, the mean length of hospital stay was similar in both groups (Table 3).

**Figure 3:**
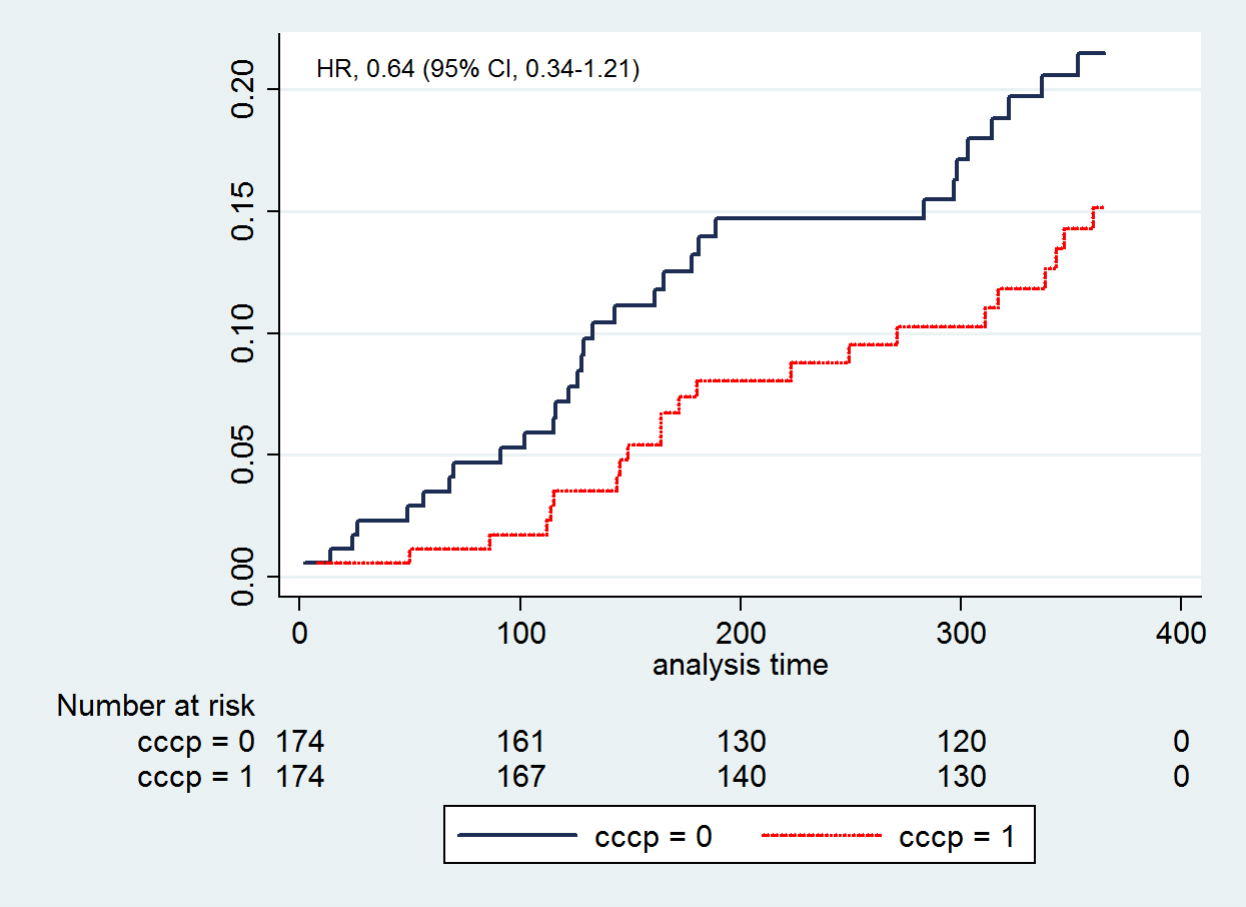
All-cause mortality.

Following up patients for an additional 8 months after program completion showed that the differential effect in hospitalization events observed at the end of the program did not persist. The rates increased among the CCCP patients but were not significantly different compared to controls (HR=1.12, 95% CI 0.90-1.41, *P*=.31). Compared with controls, mortality rates over 1-year follow-up were lower in the CCCP group, but this was also not statistically significant (HR=0.64, 95% CI 0.34-1.21, *P*=.17) (Figure 3).

**Table 3:** Hospitalization events.

Events	CCCP (n=174)	Control (n=174)	RR	*P* value
Any hospitalization, n (%)	82 (47.13)	80 (45.98)	1.03	.83
Multiple hospitalizations, n (%)	44 (25.29)	34 (19.54)	1.29	.20
Length of stay in days, mean (SD)	7 (8.92)	8 (8.83)		.92

## Discussion

### Principal Findings

This study was designed to evaluate the effect of CCCP, a heart failure remote monitoring program with objective feedback and coaching, compared with matched control patients that received usual care in the similar clinical settings, on clinical outcomes. Our findings from these retrospective analyses of medical record data suggest that compared to the control group, CCCP was associated with lower hospitalization and mortality rates over the 4-month program duration. Although not statistically significant, the mortality benefit appeared to continue even after following up for an additional 8 months after program completion (ie, 1 year from program enrollment) but hospitalizations increased over this period. We also observed that participants in the program were more likely to have multiple hospitalizations, but there was no difference in length of hospital stay.

Altogether, these findings suggest that the program was associated with reduction in hospitalization and mortality rates during the 4-month program duration and kept patients alive who probably would have died had they not been enrolled in the remote monitoring program. However, the program was associated with increased rates of hospitalizations and mortality after program completion. We speculate that the finding of increased hospitalization and mortality after the 4-month program period could be explained by the progressive nature of the disease, early disproportionate deaths of sicker controls, and also by the fact that participants had become dependent on being monitored remotely with regular access to monitoring devices and the telemonitoring nurses. Tapering of the beneficial effect of monitoring after disenrollment from the program at the end of the 4-month period could also suggest that patients had not developed sufficient self-competency to manage the disease after leaving the program. An alternative explanation for higher hospitalization rates could also be that patients had become sensitized to the early symptoms of disease decompensation due to telemonitoring education and would present earlier than patients who did not have this education.

Although mortality rates increased in the CCCP group after program completion, the overall effect was still beneficial compared to controls over the 1-year follow-up. This finding is similar to results from eight meta-analyses published between 2007 and 2013 evaluating the effect of remote monitoring on mortality [11-17]. These studies reported that compared to usual care, remote monitoring reduced mortality with overall effects ranging from 17% to 51%. The variations in these effects could be explained by the difference in type (structured telephone support vs telemonitoring), speed of feedback (rapid vs non-rapid), invasiveness (invasive vs non-invasive) of remote monitoring, duration of follow-up, study designs, and severity of disease. While a majority of the studies included in these meta-analyses evaluate the effect of remote monitoring only within the monitoring period, this evaluation further monitored participants 8 months beyond the regular 4-month program duration to evaluate the residual effects of the telemonitoring and educational intervention. While the evidence of the association of remote monitoring and reductions in mortality has been consistent over time in meta-analyses, the same effect has not been demonstrated for hospitalizations. The majority of the meta-analyses referenced above reported reductions in hospitalizations except Clarke [14], who did not find a significant reduction in all-cause hospitalization. Likewise, some recent prospective trials have also demonstrated varied effects, ranging from no effect [18-20] to reduction in hospitalizations rates by 37% [21]. Among many other reasons that may account for the incongruity between mortality and hospitalization effects, it is generally easier to assess mortality, which is a hard endpoint that is difficult to miss, unlike hospitalizations, which may be unreported, misclassified, or missed in controls.

Traditionally, remote monitoring is seen as a short-to-medium term adjunct to regular care to empower patients for self-management following hospitalization. Long-term use is not usually feasible due to cost. However, based on our findings, we speculate that increasing the duration of the program to enable patients to develop self-competency may improve outcomes. Although this may not be cost-effective for all participants, risk stratification to identify patients who will benefit from prolonged monitoring may be needed. Alternatively, because patients have built disease self-competency on the program and accompanying monitoring devices, a graduated removal of program components (ie, keeping patients on some sort of less intensive monitoring after discharge from the program) may be helpful. Less intensive monitoring following a remote monitoring program like CCCP is appealing and feasible because of the increasing availability and reducing costs of consumer-oriented monitoring devices that can be easily used by patients [22,23]. Future prospective research to evaluate optimal program monitoring duration, risk stratification to identify patients that may benefit for prolonged monitoring, and the prospects of less intensive, long-term monitoring is needed.

### Limitations

Apart from the retrospective nature of this study, it has a number of other limitations. The individual matching done in this study did not include any measure of disease severity. To ensure that patients and their matched controls were comparable at baseline, in terms of severity of disease, we evaluated and found that the rates of hospitalization were similar in both groups 120 days prior to (the CCCP patient and corresponding matched control) enrollment in the CCCP program. We also controlled for measures of disease severity (ejection fraction and NYHA classification) in our analysis. Another limitation is that any hospital admission occurring outside of the Partners’ electronic medical records were not captured in these analyses. However, this effect is minimized in the CCCP group because they were monitored daily and had more regular contact with their health care providers. On the other hand, we were more likely to have missed hospitalizations in the control group who may have out-of-system hospitalizations. We also cannot rule out unmeasured confounding from comorbidities and the fact that control patients might have received other treatments including a remote monitoring program other than CCCP. Additionally, given that the program was implemented in the setting of an academic medical center, findings from this evaluation may be generalizable only to such settings.

### Conclusions

Results from these analyses suggest that compared with usual care controls, this remote monitoring program is associated with significantly lower hospitalization rates up to 90 days and significantly lower mortality rates over 120 days of the program. However, these effects did not persist over an additional 8 months of follow-up after program completion. There is a need to evaluate the potential impact of risk stratification to determine optimal duration for remote monitoring and also the effect of less intensive, long-term remote monitoring.

## Abbreviations

CCCP: Connected Cardiac Care Program
ICD-9-CM: International Classification of Disease, Ninth Revision, Clinical Modification
MGH: Massachusetts General Hospital
NYHA: New York Heart Association
RMDR: Remote Monitoring Data Repository
RPDR: Research Patient Data Repository
